# Speech Therapy Combined With Cerebrolysin in Enhancing Nonfluent Aphasia Recovery After Acute Ischemic Stroke: ESCAS Randomized Pilot Study

**DOI:** 10.1161/STROKEAHA.124.049834

**Published:** 2025-02-17

**Authors:** Volker Homberg, Dragoș Cătălin Jianu, Adina Stan, Ștefan Strilciuc, Vlad-Florin Chelaru, Michał Karliński, Michael Brainin, Wolf Dieter Heiss, Dafin F. Muresanu, Pamela M. Enderby

**Affiliations:** Department of Neurorehabilitation, SRH Gesundheitszentrum Bad Wimpfen GmbH, Germany (V.H.).; First Division of Neurology, Department of Neurosciences-VIII, “Victor Babeș” University of Medicine and Pharmacy, Timisoara, Romania (D.C.J.).; Advanced Centre for Cognitive Research in Neuropsychiatric Pathology (NeuroPsy-Cog), Department of Neurosciences-VIII, “Victor Babeș” University of Medicine and Pharmacy, Timisoara, Romania (D.C.J.).; First Department of Neurology, “Pius Brînzeu” Emergency County Hospital, Timisoara, Romania (D.C.J.).; Department of Neuroscience, “Iuliu Hațieganu” University of Medicine and Pharmacy, Cluj-Napoca, Romania (A.S., V.-F.C., D.F.M.).; Department of Genomics, MEDFUTURE Institute for Biomedical Research, “Iuliu Hațieganu” University of Medicine and Pharmacy, Cluj-Napoca, Romania (S.S.).; Research Unit, RoNeuro Institute for Neurological Research and Diagnostic, Cluj-Napoca, Romania (A.S., S.S., V.-F.C., D.F.M.).; 2nd Department of Neurology, Institute of Psychiatry and Neurology, Warsaw, Poland (M.K.).; Department of Clinical Neurology, Danube University Krems, Austria (M.B.).; Max Planck Institute for Metabolism Research, Cologne, Germany (W.D.H.).; Department of Community Rehabilitation, School of Health and Related Research, University of Sheffield, United Kingdom (P.M.E.).

**Keywords:** aphasia, ischemic stroke, speech therapy, stroke

## Abstract

**BACKGROUND:**

Stroke-induced aphasia significantly impacts communication and quality of life. Despite the standard treatment being speech and language therapy, outcomes vary, highlighting the need for additional therapies. Cerebrolysin, a neuroprotective and neurotrophic agent, has shown potential in stroke management. This study addresses the notable gap in research about the combined use of Cerebrolysin and speech therapy, evaluating their synergistic potential in the treatment of aphasia.

**METHODS:**

The ESCAS trial (The Efficacy and Safety of Cerebrolysin in the Treatment of Aphasia After Acute Ischemic Stroke), a prospective, randomized-controlled, double-blinded study was conducted in 2 Romanian stroke centers. Participants included those with left middle cerebral artery territory ischemic stroke and nonfluent aphasia, enrolled 3 to 5 days poststroke. Inclusion criteria were right-handedness and Romanian as the mother tongue. Participants received Cerebrolysin or a placebo combined with speech and language therapy in 10-day cycles over 3 intervals, and evaluations were done at baseline, 30, 60, and 90 days respectively. The main outcome measure was Western Aphasia Battery for language function. Changes at days 30, 60, and 90 compared with baseline were quantified, and the effect estimand used was the difference in means between groups. Secondary outcome measurements were the National Institutes of Health Stroke Scale for neurological deficit, the modified Rankin Scale for global disability, and the Barthel Index for activities of daily living.

**RESULTS:**

Out of 132 enrolled patients, 123 were included in the intention-to-treat analysis, and 120 in the per-protocol analysis. Overall, both groups showed improvement at subsequent visits compared with the baseline for Western Aphasia Battery and the National Institutes of Health Stroke Scale. The Cerebrolysin group showed greater improvements in Western Aphasia Battery (visit 4 mean increase of 35.579±16.316 [95% CI, 31.289–39.869] points; *P*<0.001) compared with the placebo group (20.774±12.486 [95% CI, 17.603–23.945] points; *P*<0.001), a difference in means of 14.805 (95% CI, 9.521–20.089) points (*P*<0.001). The Cerebrolysin group also showed significant improvements (higher decreases) in National Institutes of Health Stroke Scale scores compared with the placebo group (2.085 [95% CI, 1.076–3.094] points; *P*<0.001). Safety analysis raised no concerns (number of patients with adverse events *P*=0.105, number of adverse events per patient *P*=0.134). Additionally, the Cerebrolysin group showed greater improvements in functional independence (Barthel Index) and a trend toward reduced disability (modified Rankin Scale) compared with the placebo group.

**CONCLUSIONS:**

Cerebrolysin combined with speech and language therapy offers promising potential for enhancing recovery in poststroke nonfluent aphasia. Significant improvements were observed in language and neurological deficits, underscoring the importance of adjunctive therapies in nonfluent aphasia rehabilitation. Further research with larger cohorts is needed to fully establish the efficacy of this combination therapy.

**REGISTRATION:**

URL: https://www.isrctn.com; Unique identifier: ISRCTN54581790.

Aphasia stands out as one of the most debilitating consequences of ischemic stroke.^1,2^ It not only affects the individual’s ability to communicate, but also has profound implications on their quality of life, self-esteem, and social interactions. Poststroke aphasia can manifest in various forms, from difficulty in retrieving words to a complete loss of speech, understanding, reading, or writing.^3–5^

Speech and language therapy (SLT) remains the gold standard for aphasia rehabilitation. While many patients experience some degree of spontaneous recovery in the initial weeks to months following a stroke, the benefits of SLT in enhancing this recovery and ensuring long-term improvements are well-documented.^6^ However, the degree of recovery is variable and about one-third of stroke survivors continue to live with chronic aphasia.^7^ This variability underscores the need for adjunctive treatments that can potentiate the effects of SLT and offer hope to those with persistent communication deficits.^3^

Cerebrolysin, a biological compound composed of a blend of low–molecular weight peptides and amino acids, has been investigated for its promising neuroprotective and neurotrophic properties. These properties stem from its capability to replicate the actions of endogenous neurotrophic factors, thus fostering neural protection and facilitating neurological recovery.^8^ Beneficial effects of Cerebrolysin are attributed to its ability to reduce oxidative stress, inflammation, and apoptosis in the brain.^9^ Several clinical trials have investigated the therapeutic potential of Cerebrolysin in patients with neurological conditions, including acute ischemic stroke.^10–15^ These studies have reported improvements in clinical outcomes, reduced infarct volume, and enhanced neurological recovery. Cerebrolysin has been generally well-tolerated in clinical trials and has been included in several clinical practice guidelines.^16–18^ While Cerebrolysin’s use in ischemic stroke recovery is well-established, its specific efficacy in improving language function, particularly in nonfluent aphasia, is an area that requires further exploration. A study assessing the efficacy of Cerebrolysin in patients with Broca aphasia following a first acute ischemic stroke in the left middle cerebral artery demonstrated that, when used as adjuvant therapy, Cerebrolysin can lead to significant improvements, compared with placebo.^19^ Despite these findings, our review of the literature revealed a notable lack of research specifically targeting the role of Cerebrolysin in aphasia. The scarcity of focused studies on Cerebrolysin’s role in language function recovery underscores a significant gap in current clinical knowledge.

The ESCAS trial (Efficacy and Safety of Cerebrolysin in the Treatment of Aphasia After Acute Ischemic Stroke) was conceived to bridge this knowledge gap. By evaluating the synergistic effects of Cerebrolysin and SLT, the study aimed to provide insights that could improve the therapeutic approach to nonfluent aphasia rehabilitation.

## Methods

The ESCAS study was an exploratory, prospective, randomized-controlled, double-blinded, academic study designed to assess the efficacy and safety of Cerebrolysin in combination with speech therapy compared with a placebo (saline solution) in combination with speech therapy for the treatment of nonfluent aphasia following acute ischemic stroke. All data and materials have been made publicly available at the Harvard Dataverse and can be accessed at https://doi.org/10.7910/DVN/Z8WAKW. The study was performed after drug approval by the European Medicines Agency and reimbursement in several counties worldwide to explore its effects on a wider stroke population and outcomes, hence being classified as a postmarketing surveillance study (phase 4). The study’s focus on nonfluent aphasia allowed the use of objective outcome measurements, ensuring patient compliance. No concomitant use of Cerebrolysin for motor recovery was allowed. The trial enrolled patients between June 2020 and October 2022 in 2 Romanian stroke centers (Cluj County Emergency Hospital in Cluj-Napoca, and “Pius Brînzeu” County Emergency Hospital in Timisoara), enrolling consecutive eligible patients with stroke, and was approved by the Ethics Committee of the “Iuliu Hațieganu” University of Medicine and Pharmacy (Cluj-Napoca, Romania; ref: 122/24.03.2020), as well as local hospital institutional review board. The study, as well as the accompanying clinical study protocol and statistical analysis protocol, were prospectively registered in the International Standard Randomized Controlled Trial Number with the identifier ISRCTN54581790 (https://doi.org/10.1186/ISRCTN54581790)^20^ and is reported based on the CONSORT guidelines (Consolidated Standards of Reporting Trials; Supplemental Material).^21^

### Study Inclusion Criteria

- Radiological (either computed tomography or magnetic resonance imaging) and clinical confirmation of acute ischemic stroke in the left MCA territory.
- Presence of nonfluent aphasia.
- Enrollment in the study between 3 and 5 days poststroke.
- Right-handedness.
- Daily use of Romanian as the primary language.
- Provision of signed informed consent.

### Study Exclusion Criteria

- History of symptomatic ischemic or hemorrhagic stroke.
- Severe comprehension deficits that could compromise the understanding of informed consent or instructions, such as fluent aphasias (eg, Wernicke aphasia) or global aphasias.
- History of epilepsy or electroencephalography-documented epileptic discharges.
- Severe chronic renal or liver failure, indicated by aspartate aminotransferase, alanine aminotransferase levels >4× the normal values, or creatinine levels exceeding 4 mg/dL.
- Presence of life-threatening diseases.
- Uncorrectable auditory or visual deficits that could impair testing.
- Preexisting neurodegenerative or psychiatric disease.

### Study Procedures

Upon initiation of the study, participants were allocated to 2 treatment arms and underwent their first visit, scheduled between 3 and 5 days poststroke. This baseline visit (day 0) involved the collection of participants’ demographics and medical history. Comprehensive assessments were conducted using tools such as the Western Aphasia Battery (WAB) and the National Institutes of Health Stroke Scale (NIHSS). A month into the study, specifically on day 30±3, participants returned for their second visit. During this session, evaluations were performed using the WAB, NIHSS, Barthel Index (BI), and the modified Rankin Scale (mRS). Any adverse events (AE) or severe adverse events (SAE) experienced by the participants were also meticulously documented. Two months post-baseline, on day 60±3, the third visit took place. Participants underwent similar evaluations as the previous visit, including monitoring for any AE or SAE. The fourth and last visit was on day 90±3. This final session mirrored the assessments and monitoring procedures of the second and third visits. Parallel to the study visits, participants received a structured treatment regimen. They received an intravenous infusion of 30 mL Cerebrolysin (diluted in saline, total volume 250 mL) or saline (250 mL) and underwent 1 hour of speech therapy per day for 10 days within 2-week intervals, amounting to a total of 3 treatment cycles during the periods of study days 1 to 14, 29 to 42, and 57 to 70. The total amount of SLT for both study groups was 30 hours. The therapy sessions were conducted by neuropsychologists experienced in SLT, as no additional national-level certification exists in this specific field. To ensure consistency in therapeutic approach and materials, the content and resources for speech therapy were standardized across all study centers using principles and practical applications described in Chapter IV: Assessment and Rehabilitation of the *Aphasia Handbook* by Alfredo Ardila (Florida International University, 2014). Throughout individual speech therapy sessions, patients received various worksheets to follow the entire recovery protocol and to cover a wide range of examples, enabling them to continue their recovery independently or with family support after the formal therapy sessions concluded. The intensity of the regimen was adapted to suit patient needs and the content was personalized to account for other barriers such as literacy, based on the best judgment of the therapist. Given the constraints imposed by the pandemic, some sessions were conducted remotely to minimize patient contact with therapists, especially during the March 2020 widespread lockdowns.

### Blinding and Randomization

The study was conducted under double-blind conditions, ensuring that investigators, study personnel, and patients remained unaware of treatment allocations. Given the amber color of Cerebrolysin, colored infusion lines were utilized during drug administration. Randomization was performed 1:1 in blocks of 4 and envelopes designated for each enrolled patient were handed to the study nurses responsible for preparing the infusion solution, who were excluded from other study-related procedures and were strictly instructed not to disclose any information about treatment allocation. Treatment envelopes were only opened once the patient’s initial infusion was ready. Patients who met the inclusion and exclusion criteria were assigned a random number from a pregenerated list by a selected biometrician. Based on this list, sealed, opaque randomization/emergency envelopes were distributed (1) to the study center, for instances where unblinding was necessary due to potential harm to a patient; (2) to the individual preparing the infusion; and (3) to the study coordinator. Upon opening, the date and time were recorded on the randomization/emergency envelopes, and they were signed by the individual who opened them. Any premature unblinding of the investigational products was immediately documented by the investigator and communicated to the coordinator. The entire study was unblinded after the database was closed and the analysis populations were determined.

### Evaluations

The WAB is an instrument for assessing the language function of adults with suspected neurological disorders because of a stroke, head injury, or dementia.^22^ It aids in identifying the presence, severity, and type of aphasia, while also establishing a baseline for monitoring changes during therapy, identifying key areas in the language capabilities of the patient to be improved during treatment, and inferring the location of the lesion that caused aphasia. The WAB targets English-speaking adults and teens with a neurological disorder between the ages of 18 and 89 years old. The WAB tests both linguistic skills (such as speech, fluency, auditory comprehension, reading, and writing), as well as nonlinguistic skills (such as drawing, calculation, block design, and apraxia). This study used a Romanian linguistically validated version of WAB.^23^ The Aphasia Quotient (AQ) used for our analysis was derived using the full suite of WAB assessments encompassing spontaneous speech, comprehension, repetition, and naming subtests. The AQ, which ranges from 0 to 100, serves as a comprehensive measure of overall language function, where higher scores indicate better language abilities and lower severity of aphasia. A change in AQ score is considered clinically significant when it exceeds the minimally important clinical difference threshold of 5 points, as this reflects a meaningful improvement or decline in the patient’s communicative function.^24–26^

The NIHSS is a 15-item scale that covers the level of consciousness, gaze, visual fields, facial palsy, motor functions, limb ataxia, aphasia, dysarthria and extinction, and inattention, to assess neurological deficits.^27^

The mRS a functional outcome scale measuring global outcomes.^28^ It is used for grading the outcome and the level of disability after a stroke. The mRS is a 7-point ordinal scale with a score of 0 indicative of no residual symptoms at all and the worst possible score of 6 which is assigned in case of death.

The BI is an ordinal scale used to measure performance in activities of daily living.^29,30^ Ten variables describing activities of daily living and mobility are scored, a higher number being a reflection of a greater ability to function independently following hospital discharge.

### Study End Points

The primary objective of the ESCAS study was to assess the efficacy of Cerebrolysin and speech therapy versus placebo (saline solution) and speech therapy at 30, 60, and 90 days after baseline. Efficacy was evaluated using the WAB (Romanian translated version) scores at each time point. The primary outcome measure was the change in WAB-AQ score from baseline to each follow-up time point (30, 60, and 90 days).

The secondary objectives of the ESCAS study included: (1) to assess the efficacy of Cerebrolysin and speech therapy versus placebo (saline solution) and speech therapy at 30, 60, and 90 days after baseline using measures of motor, neurological, and global functional outcome. These outcomes were evaluated using the NIHSS, BI, and mRS scores at each time point; (2) to evaluate the safety of Cerebrolysin and speech therapy versus placebo (saline solution) and speech therapy at 30, 60, and 90 days after baseline. Safety was assessed by comparing the incidence of AE and SAE between the 2 treatment groups. Additionally, we measured specific cardiovascular (such as, but not limited to stroke, myocardial infarction, atherosclerosis, vascular stenosis, as well as their recurrence), hematologic (including anemia and vitamin B9 or B12 deficiency), renal system (including hyperuremia, hyperuricemia, and urinary tract infections) and metabolic (including dyslipidemia, diabetes, and atherosclerosis) related AE. The secondary outcome measures were focused on the assessment of changes in the NIHSS scores from the baseline to each follow-up point (30, 60, and 90 days). Notably, evaluations of the mRS and BI commenced 30 days post-baseline, with subsequent changes measured at each follow-up. The study also monitored the incidence of AEs and SAEs.

### Study Populations

The per-protocol (PP) population included all trial participants, who adhered to the study protocol, received the medication corresponding to the group they were assigned to after randomization, had at most minimal protocol deviations, and no missing values for total scores of WAB and NIHSS at visits 2, 3, or 4, or missing values for mRS and BI at visits 3 or 4. The intention-to-treat (ITT) population included all trial participants who were registered in the trial and were randomized, regardless of their subsequent adherence to the protocol or premature discontinuation. This analysis set was used to assess the efficacy of the studied treatment, considering patients not adhering to the protocol (noncompliance, dropouts, SAEs, unforeseen events), thus better representing the expected results of the treatment in clinical practice. The same statistical procedures were applied to both PP and ITT populations. The Safety population included all trial participants, who were registered in the trial and received at least 1 dose of treatment, regardless of their subsequent adherence to the protocol or premature discontinuation. Unlike ITT, the safety population analysis focused on AEs and SAEs caused by the treatment, thus evaluating the safety-related parameters of the products.

### Sample Size Estimation

Preceding the clinical trial, we determined the sample size for the ESCAS study through a power analysis using G*Power, 3.1.9.7.^31^ Assuming a medium effect size *d*=0.5, an alpha error of *α*=0.05 and *β*=0.2, with an allocation ratio of 1 for Wilcoxon-Mann-Whitney *U* test between 2 groups, we obtained a sample size of 53 patients per group. To provide a margin of error for situations such as patient withdrawal, SAEs, or protocol violations, we have decided to recruit at least 120 patients in total for our study, 60 for each arm.

### Data Analysis

We used Microsoft Excel 2019 (Microsoft Corporation, Redmond, WA), for data preparation and cleanup and R, v. 4.3.1 and v. 4.4.2 (R Core Team, Vienna, Austria) and RStudio (Posit Software, PBC, Boston, MA) for data analysis, loading the ggplot2,^32^ readxl,^33^ xlsx,^34^ MANOVA.RM,^35^ and psych^36^ libraries. For comparing numeric values of paired samples, differential values for each pair of values were computed, and a Wilcoxon signed-rank test with null hypothesis location=0 was used. For comparing numeric values of unpaired samples, Wilcoxon rank-sum tests with a null hypothesis of location difference equal to 0 were performed.

Given the use of multiple tests, we used Bonferroni correction to adjust *P* values. For WAB-AQ and NIHSS, *P* values of comparisons between baseline and subsequent visits were adjusted by a factor of 6, and *P* values of comparisons between treatment groups regarding change from baseline were adjusted by a factor of 3. For mRS and BI, the *P* value of comparisons between visits 2 and 3, respectively 2 and 4, were adjusted by a factor of 4, and the *P* value comparisons between scores at visits 2, 3, and 4 respectively were adjusted by a factor of 3.

For comparing the difference in prevalences of variants of 1 dichotomous or nominal variable, among the groups of another dichotomous or nominal variable (ie, testing the association between 2 dichotomous/nominal variables), the χ^2^ test was used or, where its assumptions were violated (mainly due to a small number of patients in any group), the Fisher exact test was used. Where applicable, the 2-tail *P* value was reported. The type 1 error was assumed to be *α*=0.05, and as such, results were considered statistically significant for *P*<0.05. The primary estimand for treatment effect was the difference in mean change in WAB-AQ score from baseline, between the Cerebrolysin group and placebo group at days 30, 60, and 90 respectively. Additionally, Cohen *d* and its associated CI were computed, using the package psych. The full statistical analysis protocol was published before the database unblinding.^20^

Additionally, in the per-protocol sample, we used the repeated measures function, which provides robust, resampling-based mixed-models ANOVA that is not dependent upon the normality and homoscedasticity of the data to evaluate the differences in clinical scores due to visit number (as within-subjects variable), treatment (as between-subjects variable), as well as their interaction. The function was executed with 10 000 iterations (the default value), without parallelization, and with seed 19 990 304, returning *P* values up to 10 decimal places. We reported the *P* values obtained through resampling.

## Results

**Figure 1.**
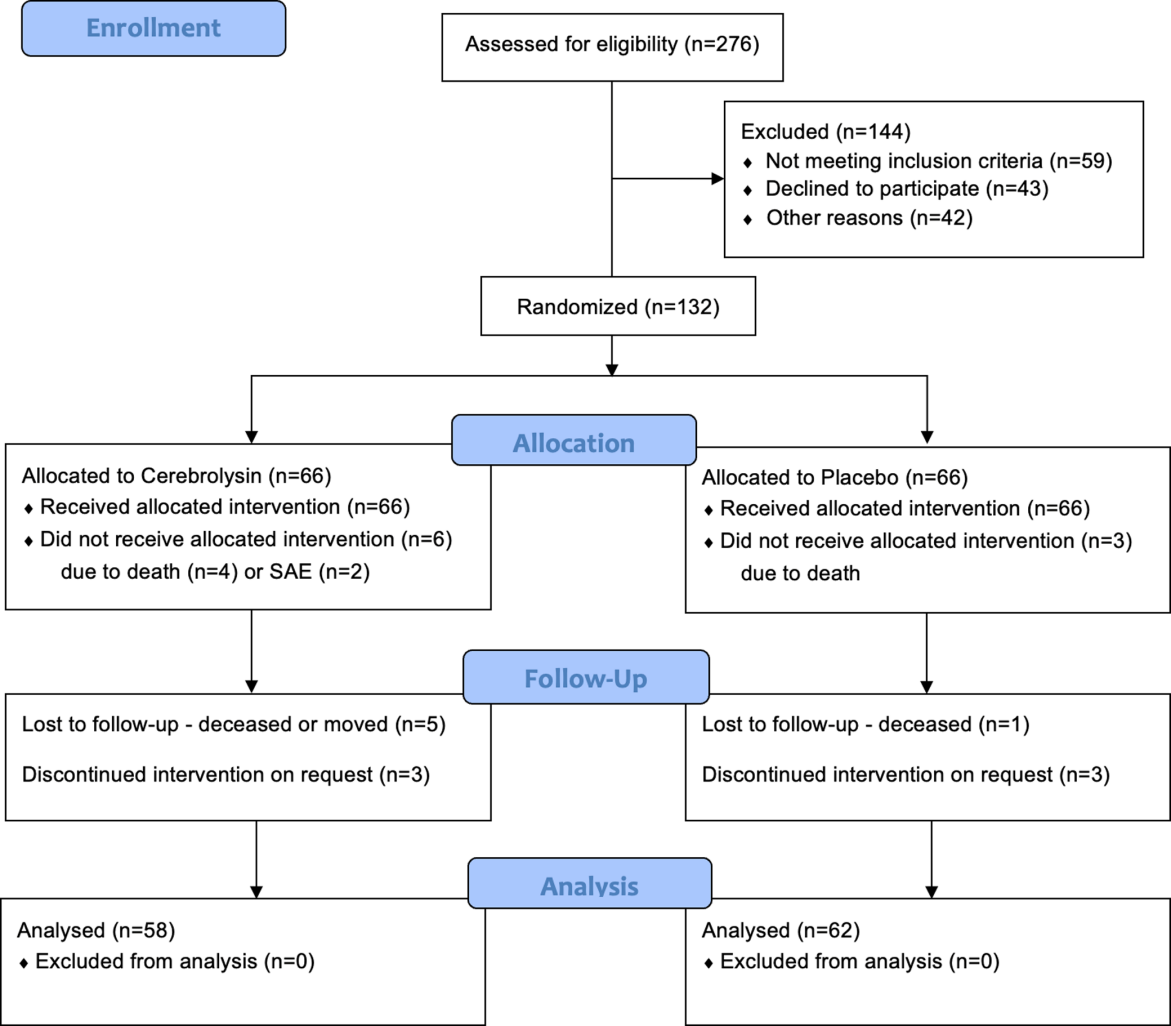
**CONSORT (Consolidated Standards of Reporting Trials) flow diagram.** SAE indicates severe adverse event.

The study enrolled 132 adult patients between June 2020 and October 2022 when a sufficient sample size was reached, of which 123 patients meeting criteria for the ITT population (visit 2: Cerebrolysin n=61, placebo n=63; visit 3: Cerebrolysin n=59, placebo n=63; visit 4: Cerebrolysin n=58, placebo n=62) and 120 for the PP population (Cerebrolysin n=58, placebo n=62). A flow diagram of per-protocol patient enrollment is available in Figure 1. We report group difference analysis performed on the PP population, which has registered nearly identical results as the ITT population, available in Supplemental Material (Tables S1 through S4).

Demographic characteristics and baseline outcome measures highlighting good baseline comparability are available in Table 1.

**Table 1.**
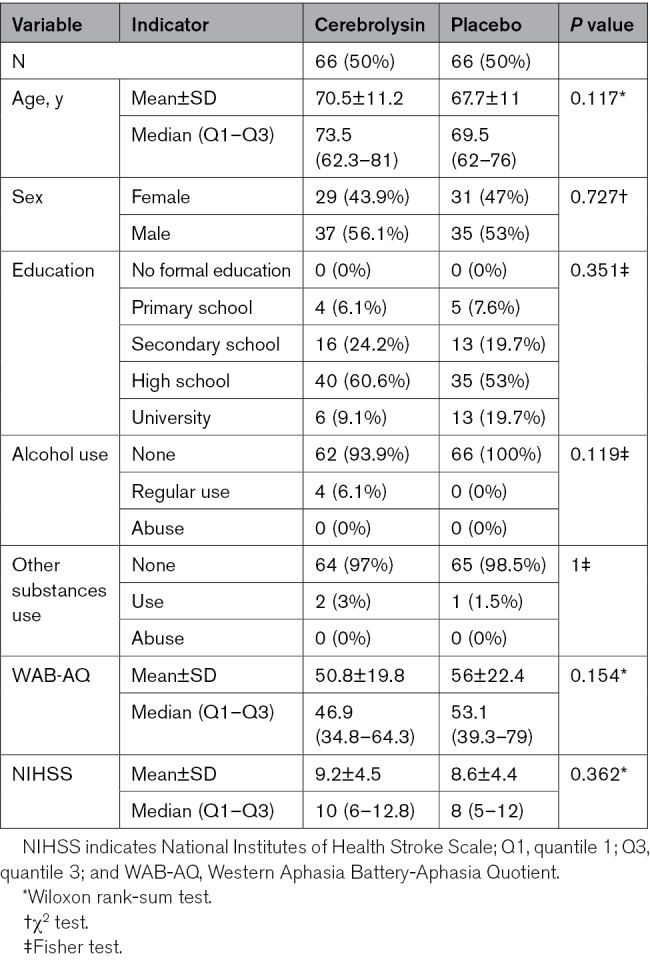
Demographic Characteristics and Baseline Values for Trial Arms

### WAB-AQ Baseline Differences

For each patient, we analyzed the differences between WAB values at subsequent visits and WAB values at baseline. PP results are presented (more analyses available in Tables S5 through S8), with ITT data available in Supplemental Material (Tables S1 through S4). For the second visit, the Cerebrolysin group showed a mean increase from baseline of 17.366±10.303 (95% CI, 14.567–20.075), statistically significant compared with baseline (*P*<0.001); the placebo group also demonstrated a mean increase from baseline of 10.266±9.688 (95% CI,7.806–12.726; *P*<0.001), which was lower than the Cerebrolysin group with a difference between averages of 7.099 (95% CI, 3.478–10.721) points (*P* value adjusted for multiple comparisons; *P*<0.001). Cohen *d* for the difference in improvements between groups was equal to 0.717 (95% CI, 0.346–1.085), showing a medium to large effect of Cerebrolysin combined with speech therapy in recovery compared with placebo.

At the third visit, the Cerebrolysin group had a mean increase from baseline of 28.166±13.601 (95% CI, 24.589–31.742) points (*P*<0.001). The placebo group also had an increase of 14.798±16.231 (95% CI, 10.676–18.92) points (*P*<0.001), with a statistically significant difference between groups regarding mean score increases of 13.368 (95% CI, 7.966–18.77; *P*<0.001). Cohen *d* was equal to 0.898 (95% CI, 0.52–1.272), showing a large effect size of Cerebrolysin compared with placebo.

**Figure 2.**
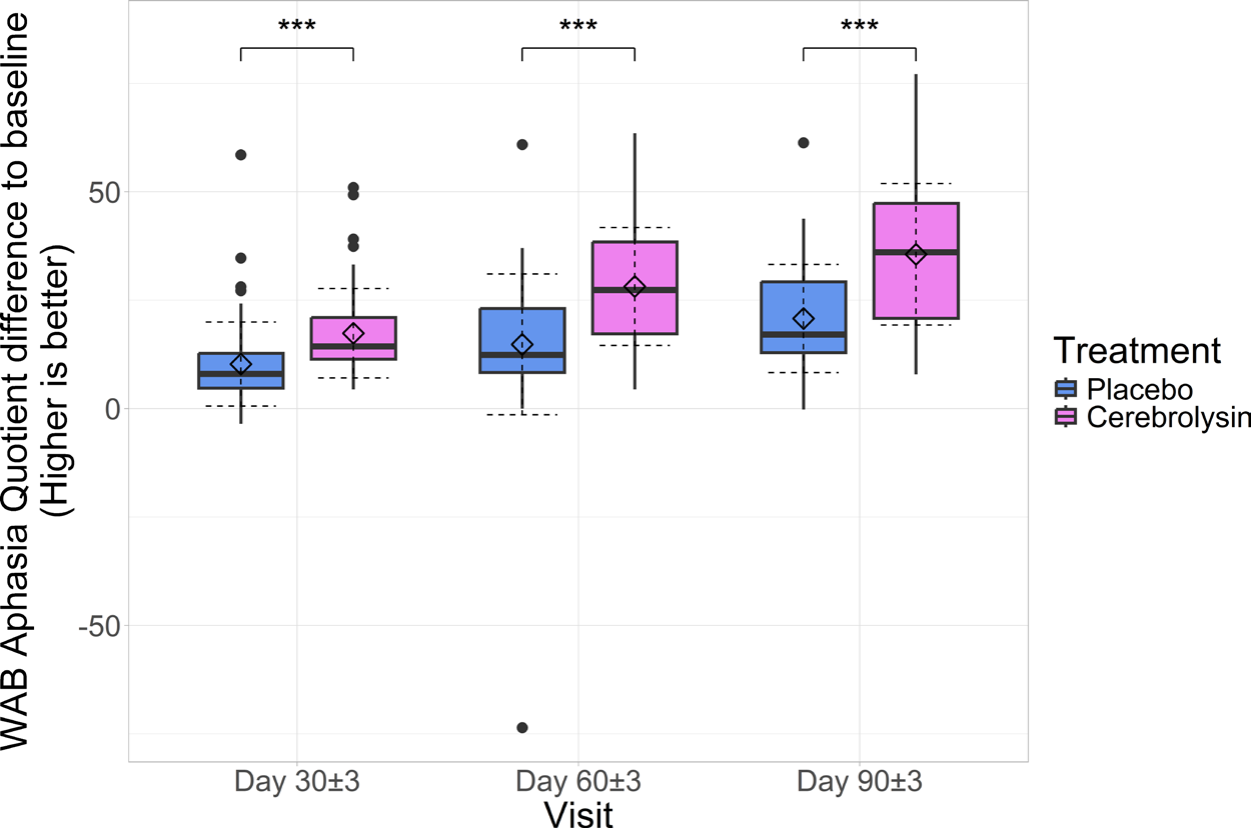
**Boxplots of Western Aphasia Battery-Aphasia Quotient (WAB-AQ) baseline differential across study visits; horizontal lines: medians, diamonds: means; solid whiskers: range (excluding outliers marked with points); dashed error lines: SD relative to the means.** Accolades represent Wilcoxon rank-sum test between groups, adjusted for multiple comparisons; ***P<0.001.

By the fourth visit, the Cerebrolysin group achieved a mean increase of 35.579±16.316 (95% CI, 31.289–39.869; *P*<0.001), and the placebo group showed a mean increase of 20.774±12.486 (95% CI, 17.603–23.945; *P*<0.001), which was statistically significantly lower compared with the Cerebrolysin group with 14.805 (95% CI, 9.521–20.089) points (*P*<0.001; Figure 2). Cohen *d* was equal to 1.032 (95% CI, 0.649–1.412), signifying a large effect size of Cerebrolysin compared with placebo.

### NIHSS-Baseline Differential Analysis

At the second visit, NIHSS scores showed statistically significant decreases both in the Cerebrolysin group (3.069±1.918 [95% CI, 2.565–3.573]; *P*<0.001) as well as in the placebo group (2.274±1.32 [95% CI, 1.939–2.609]; *P*<0.001). The decreases tended to be greater in the Cerebrolysin group, of 0.795 (95% CI, 0.194–1.395) points (*P*=0.058 adjusted for multiple comparisons). Cohen *d*=0.49 (95% CI, 0.125–0.852) for the comparison of decreases between treatment groups, showed a medium effect size of Cerebrolysin compared with placebo.

At the third visit, the Cerebrolysin group showed a greater decrease in NIHSS scores (4.914±2.786 [95% CI, 4.181–5.646]) compared with the placebo group (3.306±1.807 [95% CI, 2.848–3.765]), with the difference between groups being statistically significant (1.607 [95% CI, 0.75–2.465]; *P*=0.001). Cohen *d* was 0.695 (95% CI, 0.325–1.062), showing a medium to large effect of Cerebrolysin compared with placebo.

**Figure 3.**
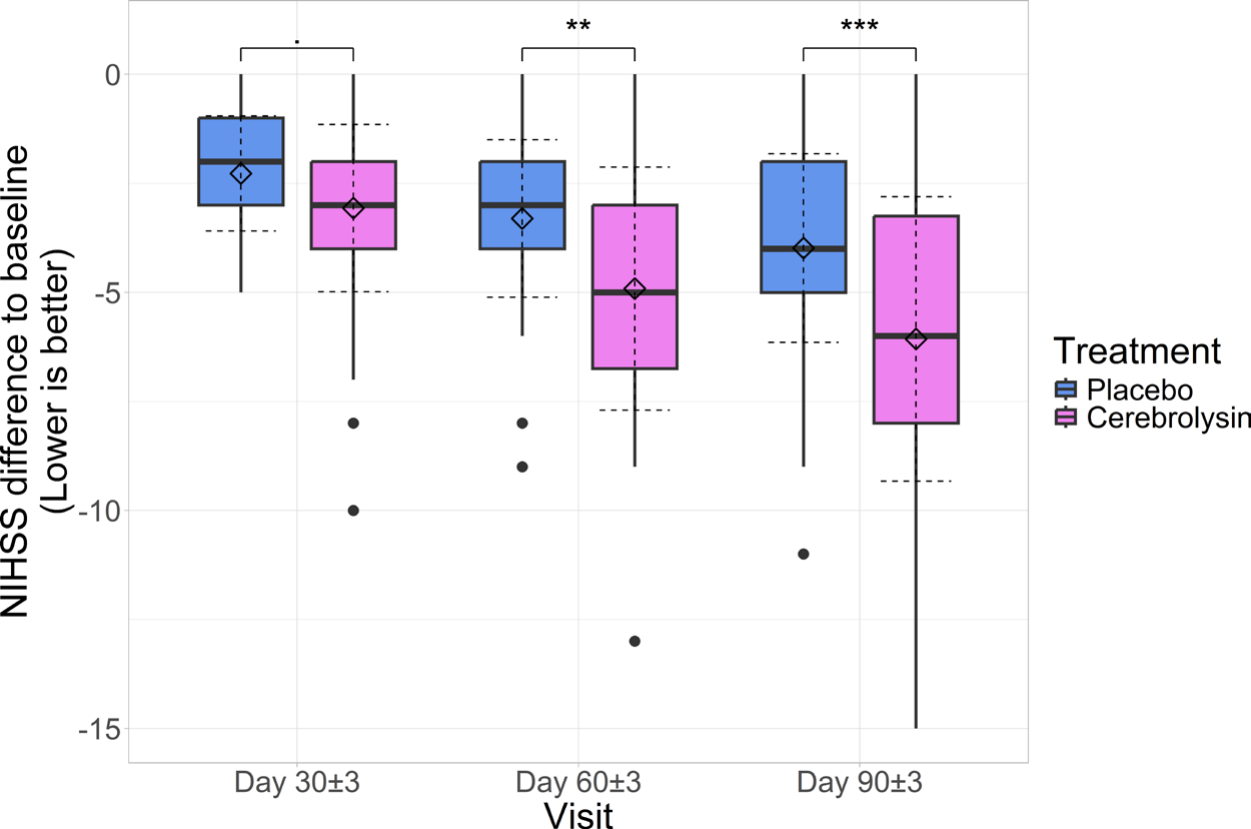
**Boxplots of National Institutes of Health Stroke Scale (NIHSS) baseline differential across study visits; horizontal lines: medians, diamonds: means; solid whiskers: range (excluding outliers marked with points); dashed error lines: SD relative to the means.** Accolades represent Wilcoxon rank-sum test between groups, adjusted for multiple comparisons; 0.05≤*P*<0.1; ***P<0.001.

By the fourth visit, there were statistically significantly higher decreases in the Cerebrolysin group (6.069±3.26 [95% CI, 5.212–6.926]) compared with the placebo group (3.984±2.161 [95% CI, 3.435–4.533]), with the difference between groups being statistically significant (2.085 [95% CI, 1.076–3.094]; *P*<0.001). Cohen *d* was equal to 0.765 (95% CI, 0.393–1.135), highlighting a medium to large effect size of Cerebrolysin compared with placebo (Figure 3).

### mRS: Measurement at Visits 2, 3, and 4 Analysis

At the second visit, the mean score in the Cerebrolysin group was 2.931±1.153, while in the placebo group, it was 2.839±1.176, with no statistically significant differences between groups (difference between means, 0.092 [95% CI, −0.329 to 0.513]; *P*>0.999 adjusted for multiple comparisons). Cohen *d*=0.08 (95% CI, −0.278 to 0.438), showing no effect of Cerebrolysin compared with placebo in absolute mRS values.

At the third visit, the mean score for the Cerebrolysin group was 2.345±1.101, while in the placebo group, it was 2.532±1.224, with no statistically significant differences between groups (0.187 [95% CI, −0.233 to 0.608]; *P*>0.999). Cohen *d*=0.162 (95% CI, −0.197 to 0.52), showing no effect of Cerebrolysin compared with placebo in absolute mRS values.

By the last visit, the mean score for the Cerebrolysin group was 1.759±1.048, while the mean score for the placebo group was 2.258±1.28, with a tendency for scores in the Cerebrolysin group to be lower compared with the placebo group (0.499 [95% CI, 0.078–0.921]; *P*=0.081). Cohen *d*=0.429 (95% CI, 0.066–0.791), showing a small to medium effect size of Cerebrolysin compared with placebo in absolute mRS values.

**Figure 4.**
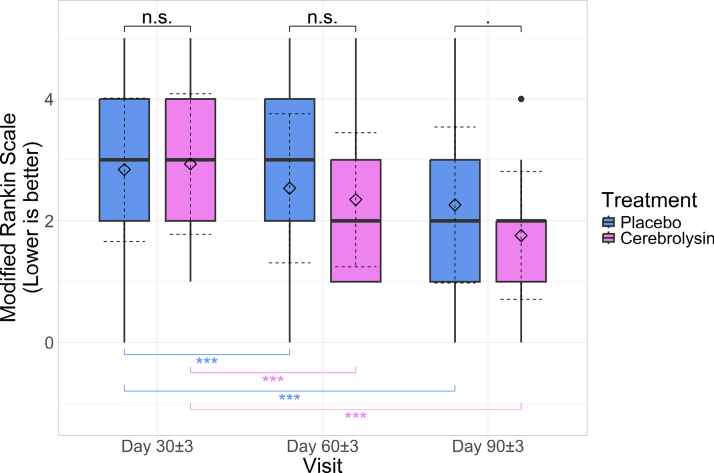
**Boxplots of modified Rankin Scale (mRS) scores across study visits; horizontal lines: medians, diamonds: means; solid whiskers: range (excluding outliers marked with points); dashed error lines: SD relative to the means.** Upper accolades represent Wilcoxon rank-sum test between groups, adjusted for multiple comparisons, while lower accolades represent Wilcoxon signed-rank test between visits for each group; 0.05≤*P*<0.1; ***P<0.001.

Of note, for both groups, values at third and fourth visits were significantly decreased compared with the second visit (*P*<0.001 adjusted for multiple comparisons; Figure 4), and the mean decreases were greater in the Cerebrolysin group compared with the placebo group both for visit 3 (0.28 [95% CI, 0.093–0.466]; *P*=0.01) and visit 4 (0.592 [95% CI, 0.315–0.868]; *P*<0.001). Cohen *d* for comparing mRS decreases between groups was equal to 0.548 (95% CI, 0.182–0.912; medium effect size) and −0.79 (95% CI, 0.416–1.16; large effect size), for visits 3 and 4 respectively.

### BI: Measurement at Visits 2, 3, and 4 Analysis

At the second visit, the mean score in the Cerebrolysin group was 64.31±26.448, while the mean score in the placebo group was 64.355±28, with no statistically significant differences between groups (difference between means, −0.044 [95% CI, −9.887 to 9.798]; *P*>0.999 adjusted for multiple comparisons). Cohen *d*=0.002 (95% CI, −0.356 to 0.36), showing no effect differences between groups.

At the third visit, the mean score for the Cerebrolysin group was 74.052±22.95, while in the placebo group, it was 70.323±25.299, with no statistically significant differences between groups (3.729 [95% CI, −4.994 to 12.453]; *P*>0.999). Cohen *d*=0.155 (95% CI, −0.203 to 0.514), showing no effect difference between groups.

At the last visit, the mean score for the Cerebrolysin group was 82.586±19.108, while the mean score for the placebo group was 74.032±24.24, with no statistically significant differences between groups (8.554 [95% CI, 0.687–16.421]; *P*=0.153). Cohen *d*=0.394 (95% CI, 0.031–0.754), showing a small effect size for Cerebrolysin compared with placebo for absolute BI values.

**Figure 5.**
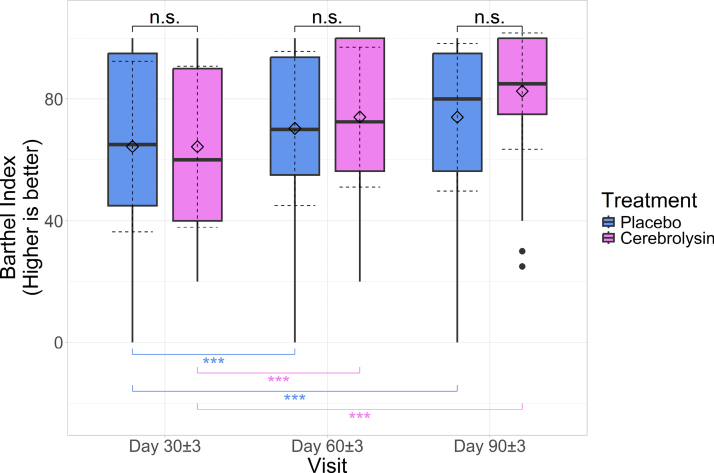
**Boxplots of Barthel Index (BI) scores across study visits; horizontal lines: medians, diamonds: means; solid whiskers: range (excluding outliers marked with points); dashed error lines: SD relative to the means.** Upper accolades represent Wilcoxon rank-sum test between groups, adjusted for multiple comparisons, while lower accolades represent Wilcoxon signed-rank test between visits for each group; ***P<0.001.

Notably, in both groups, values at the third and fourth visits were significantly decreased compared with the second visit (*P*<0.001 adjusted for multiple comparisons; Figure 5), and the mean increases were larger in the Cerebrolysin group compared with the placebo group both for visit 3 (3.774 [95% CI, 0.91–6.637]; *P*=0.015) and visit 4 (8.598 [95% CI, 3.711–13.486]; *P*=0.004). The differences between groups in increases in BI values at visits 3 and 4 compared with the second visit had Cohen *d* equal to 0.482 (95% CI, 0.117–0.844; medium effect size for Cerebrolysin compared with placebo) and 0.65 (95% CI, 0.282–1.017; medium to large effect size for Cerebrolysin compared with placebo), respectively.

Detailed analyses, including data presented as median and interquartile range, mean and SD, range, and the statistical tests applied, along with the ITT population analysis, are available in Supplemental Material (Tables S1 through S8).

### Resampling-Based MANOVA Model

Resampling-based mixed-models ANOVA was conducted to evaluate the effects of Cerebrolysin across clinical scales in the PP population (Table 2). Given the nonnormal distribution of the data, resampling-based ANOVA was chosen for its robustness in handling complex data distributions.

**Table 2.**
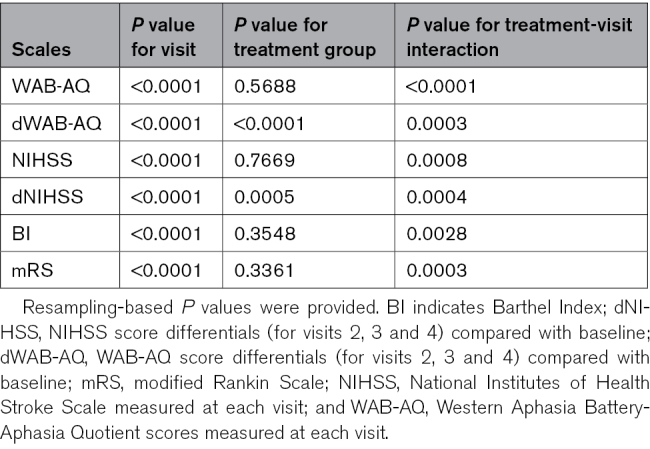
Results of MANOVA Analysis for Clinical Scales Used

Regarding the primary outcome of this study (WAB-AQ), when analyzing the raw scores from the 4 visits, there were statistically significant differences between the visits (Wald-type statistic [WTS]=454.407, df=3, *P*<0.001; ANOVA-type statistic [ATS]=252.908, df1=2.061, df2=∞, *P*<0.001; resampling-based *P*<0.001), and also there were statistically significant differences related to the (visit-group) interaction term (WTS=31.386, df=3, *P*<0.001; ATS=19.768, df1=2.061, df2=∞, *P*<0.001; resampling-based *P*<0.001). Still, there were no statistically significant differences between treatment groups (WTS=0.327, df=1, *P*=0.567; ATS=0.327, df1=1, df2=153.003, *P*=0.568; resampling-based *P*=0.569). Although the main effect of treatment alone was not significant, the significant interaction indicates that the benefits of Cerebrolysin on aphasia, as measured by the WAB, become more pronounced over time.

When analyzing the differences between WAB-AQ scores at subsequent visits compared with the baseline, there were statistically significant differences between visits (WTS=238.11, df=2, *P*<0.001; ATS=125.34, df1=1.936, df2=∞, *P*<0.001; resampling-based *P*<0.001), between groups (WTS=28.423, df=1, *P*<0.001; ATS=28.423, df1=1, df2=151.93, *P*<0.001; resampling-based *P*<0.001), as well as a statistically significant interaction between treatment group and visit (WTS=17.699, df=2, *P*<0.001; ATS=10.188, df1=1.936, df2=∞, *P*<0.001; resampling-based *P*<0.001).

### Safety Analysis

Regarding AE, there was no significant difference in the number of patients experiencing SAEs between the 2 groups (*P*=0.381). For general AEs, 43.9% of patients in the Cerebrolysin group and 30.3% in the placebo group reported events, although the difference was not statistically significant (*P*=0.105). The mean number of AEs per patient was 0.894 in the Cerebrolysin group and 0.636 in the placebo group (no difference between the number of AEs experienced by each patient between groups; *P*=0.134). Specifically, for AE categories such as neurological, psychiatric, respiratory, immune-related, and ophthalmic, both groups showed similar rates with no significant differences. The highest differences in AEs reported between groups were seen in the gastrointestinal category (4.5% of the Cerebrolysin group reported AEs, while none were reported in the placebo group; *P*=0.244) and in the osteoarticular category (no AEs were reported in the Cerebrolysin group, while 6.1% of the patients in the placebo group reported events; *P*=0.119). Additional safety information is available in the Table S9.

## Discussion

The ESCAS study sought to evaluate the efficacy and safety of Cerebrolysin in combination with speech therapy compared with a placebo (saline solution) in combination with speech therapy for the treatment of nonfluent aphasia following acute ischemic stroke. This approach draws upon findings from a study by Jianu et al, which investigated Cerebrolysin’s role in patients with Broca aphasia following an acute ischemic stroke of the left middle cerebral artery. The study observed significant improvements in language functions as evidenced by the WAB scores, highlighting Cerebrolysin’s benefit over placebo when used as an adjunct treatment.^19^ The MANOVA analysis provides evidence that the treatment effect of Cerebrolysin evolves over time. This is particularly evident in the significant interaction effects observed for WAB, NIHSS, and mRS scales. The findings suggest that Cerebrolysin accelerates recovery in a time-dependent manner, potentially making it a valuable adjunct in poststroke rehabilitation.

When looking at our results, the demographic characteristics and baseline outcome measures revealed good baseline comparability between the Cerebrolysin and placebo groups, ensuring that any observed differences in outcomes could be attributed to the interventions rather than baseline disparities.

The primary outcome measure in this study was the change in WAB scores from baseline to each follow-up time point at 30, 60, and 90 days. The results demonstrated significant improvements in the WAB-AQ for both the Cerebrolysin and placebo groups at each time point compared with baseline. These findings indicate that utilizing speech therapy, as part of the standard care, led to notable improvements in nonfluent aphasia over time. However, it is important to note that the improvements observed in the Cerebrolysin group were consistently significantly higher than those in the placebo group, with large numeric differences. This provides direct evidence supporting the addition of Cerebrolysin to SLT in the poststroke nonfluent aphasia neurorehabilitation regimen.

Secondary outcome measures, including the NIHSS, BI, and mRS, were also assessed to evaluate motor, neurological, and global functional outcomes. Similar to the WAB scores, the placebo group showed significant improvements in these measures at each time point compared with baseline, indicating that speech therapy had a positive impact on overall recovery. Still, significantly higher improvements were seen across all measurements in the Cerebrolysin group compared with those in the placebo group, thus further supporting the role of Cerebrolysin in the functional recovery of the poststroke patient.

The safety analysis included the assessment of AE and SAE in both treatment groups. No specific safety concerns related to Cerebrolysin were identified during the study. The incidence of AEs and SAEs was similar between the Cerebrolysin and placebo groups, suggesting that the addition of Cerebrolysin to speech therapy did not result in an increased risk of AE, adding to the existing body of evidence available on the safety of Cerebrolysin.^14,17,18,37^

The results of this study provide valuable insights into the potential benefits of adding Cerebrolysin to speech therapy for the rehabilitation of poststroke nonfluent aphasia. The trends observed suggest that Cerebrolysin may have a positive impact on rehabilitation. These findings warrant further investigation in larger studies with a longer follow-up period to determine whether Cerebrolysin can produce statistically significant improvements in patient outcomes. Also, while we observed improvements in the AQ scores within the placebo groups, it is important to recognize that spontaneous neurological recovery following a stroke could also lead to these positive outcomes and they cannot be solely attributed to speech therapy.

It is essential to acknowledge some limitations of this study, which could impact the generalizability of the results. The sample size, although determined through a rigorous power analysis, may have limited the ability to detect small but clinically still meaningful differences between the treatment groups. Another acknowledged limitation of our study is the lack of control for certain interindividual variables, such as lesion volume, stroke severity, and cognitive impairment, which may have introduced additional variability into our study results.

In conclusion, the ESCAS trial represents an important step in exploring Cerebrolysin as an adjunctive therapy, for nonfluent aphasia rehabilitation following acute ischemic stroke. The trends toward improved outcomes with Cerebrolysin suggest the need for larger clinical trials to confirm our results. Aphasia remains a challenging condition and finding additional therapies that can enhance recovery and quality of life for affected individuals is of paramount importance. Future studies with larger sample sizes, longer follow-up durations, and a more stringent approach in the selection of participants may provide more definitive answers regarding the efficacy of Cerebrolysin in nonfluent aphasia rehabilitation.

## Article Information

### Sources of Funding

The study was funded academically by the nonprofit Foundation of the Society for the Study of Neuroprotection and Neuroplasticity (SSNN) and the Foundation of the Study of Nanoneurosciences and Neuroregeneration (FSNANO).

### Disclosures

Dr Mureșanu discloses major financial activities (travel/accommodation/meeting expenses) with FSNANO, as well as being a principal investigator in the C-REGS II (Cerebrolysin Registry Study in Stroke) and the CARS (Cerebrolysin and Recovery after Stroke) I trial, funded by EVER Neuro Pharma, the producer of Cerebrolysin, and a principal investigator in the CAPTAIN II (Cerebrolysin Trial in Neuroprotection and Neurorecovery After Traumatic Brain Injury), CAPTAIN rTMS (Cerebrolysin and Repetitive Transcranial Magnetic Stimulation [rTMS] in Patients With Traumatic Brain Injury), CODEC (A Randomized, Placebo-Controlled, Double-Blind Trial to Asses the Effficacy and Safety of Cerebrolysin in the Treatment of Post-Stroke Cognitive Decline), ESCAS (Efficacy and Safety of Cerebrolysin in the Treatment of Aphasia After Acute Ischemic Stroke), and C-RETURN (Efficacy and Safety of Cerebrolysin for Neurorecovery After Moderate-Severe Traumatic Brain Injury) clinical trials, funded academically. Dr Brainin was a principal investigator in the CASTA trial (Cerebrolysin in Patients With Acute Ischemic Stroke in Asia) and has previously received honoraria as a speaker and as a consultant for EVER Neuro Pharma. Dr Stan discloses being an investigator in the CODEC study on Cerebrolysin in stroke, funded academically. Dr Heiss was an investigator in the CASTA, CARS I, and C-REGS II studies. Dr Homberg was an investigator in the CAPTAIN trial series. Dr Karliński has received honoraria as a speaker for EVER NeuroPharma. The other authors report no conflicts.

### Supplemental Material

Tables S1–S9

R Source Code for Demographics and AE Analyses

R Source Code for Outcome Variables Analyses

CONSORT Checklist
